# The Metabolic Redox Regime of Pseudomonas putida Tunes Its Evolvability toward Novel Xenobiotic Substrates

**DOI:** 10.1128/mBio.01512-18

**Published:** 2018-08-28

**Authors:** Özlem Akkaya, Danilo R. Pérez-Pantoja, Belén Calles, Pablo I. Nikel, Víctor de Lorenzo

**Affiliations:** a Department of Molecular Biology and Genetics, Faculty of Sciences, Gebze Technical University, Kocaeli, Turkey; b Programa Institucional de Fomento a la Investigación, Desarrollo e Innovación, Universidad Tecnológica Metropolitana, Santiago de Chile, Chile; c Systems and Synthetic Biology Program, Centro Nacional de Biotecnología, Madrid, Spain; d Novo Nordisk Foundation Center for Biosustainability, Technical University of Denmark, Kongens Lyngby, Denmark; University of Washington

**Keywords:** NADPH oxidases, *Pseudomonas putida*, reactive oxygen species, biodegradation, dinitrotoluene, evolution, oxidative stress

## Abstract

During evolution of biodegradation pathways for xenobiotic compounds involving Rieske nonheme iron oxygenases, the transition toward novel substrates is frequently associated with faulty reactions. Such events release reactive oxygen species (ROS), which are endowed with high mutagenic potential. In this study, we evaluated how the operation of the background metabolic network by an environmental bacterium may either foster or curtail the still-evolving pathway for 2,4-dinitrotoluene (2,4-DNT) catabolism. To this end, the genetically tractable strain Pseudomonas putida EM173 was implanted with the whole genetic complement necessary for the complete biodegradation of 2,4-DNT (recruited from the environmental isolate *Burkholderia* sp. R34). By using reporter technology and direct measurements of ROS formation, we observed that the engineered P. putida strain experienced oxidative stress when catabolizing the nitroaromatic substrate. However, the formation of ROS was neither translated into significant activation of the SOS response to DNA damage nor did it result in a mutagenic regime (unlike what has been observed in *Burkholderia* sp. R34, the original host of the pathway). To inspect whether the tolerance of P. putida to oxidative challenges could be traced to its characteristic reductive redox regime, we artificially altered the NAD(P)H pool by means of a water-forming, NADH-specific oxidase. Under the resulting low-NAD(P)H status, catabolism of 2,4-DNT triggered a conspicuous mutagenic and genomic diversification scenario. These results indicate that the background biochemical network of environmental bacteria ultimately determines the evolvability of metabolic pathways. Moreover, the data explain the efficacy of some bacteria (e.g., pseudomonads) to host and evolve with new catabolic routes.

## Abstract (precis)

Some environmental bacteria evolve with new capacities for the aerobic biodegradation of chemical pollutants by adapting preexisting redox reactions to novel compounds. The process typically starts by cooption of enzymes from an available route to act on the chemical structure of the substrate-to-be. The critical bottleneck is generally the first biochemical step, and most of the selective pressure operates on reshaping the initial reaction. The interim uncoupling of the novel substrate to preexisting Rieske nonheme iron oxygenases usually results in formation of highly mutagenic ROS. In this work, we demonstrate that the background metabolic regime of the bacterium that hosts an evolving catabolic pathway (e.g., biodegradation of the xenobiotic 2,4-DNT) determines whether the cells either adopt a genetic diversification regime or a robust ROS-tolerant status. Furthermore, our results offer new perspectives to the rational design of efficient whole-cell biocatalysts, which are pursued in contemporary metabolic engineering.

## INTRODUCTION

Environmental bacteria that catabolize xenobiotic pollutants (existing only since the onset of synthetic chemistry) offer a unique opportunity to inspect the rules that govern the evolution of metabolic networks (1, 2). Unlike resistance to antibiotics, which can be caused by mutations modifying the target or by evolving just one protein (3–6), new catabolic phenotypes require multiple changes in the protein complement of the pathway along with other functions in the host that tune the activity of the novel route according to the background biochemical network (7–10). Among the microorganisms known to host aerobic routes for catabolism of typical industrial pollutants, e.g., chloroaromatic (11, 12) and nitroaromatic chemicals (13, 14), *Pseudomonas* species stand out as recurrent hosts of catabolic routes that enable growth on such unusual chemicals (15–20). This state of affairs raises two related questions. The first question is why new pathways evolve preferentially in this bacterial domain and not so much in other species. The second question is how an evolutionary solution to the novel metabolic challenge remains in the same bacterial domain rather than propagation into other prokaryotic hosts (15). The biochemical reactions at stake often involve redox modifications on the substrate, such as mono- or dioxygenations executed by Rieske nonheme iron oxygenases (21–23). Owing to their mechanism of action, when such enzymes act on substrates-to-be that do not fit well in the active enzyme center, reactive oxygen species (ROS) are released (24–27). This phenomenon is due to a suboptimal kinetic scenario in which the substrate leaves the active pocket unscathed before the Fe-activated oxygen molecule can attack the aromatic structure (28, 29). Uncoupled reactions of this sort result in considerable redox stress in the host (30) and—at least in some cases—DNA damage and acquisition of a DNA mutagenic regime (31, 32). The interplay between faulty redox reactions, ROS formation, and DNA damage has been previously characterized in strain *Burkholderia* sp. R34. This environmental bacterium degrades (if with difficulties) 2,4-dinitrotoluene (2,4-DNT), an archetypal xenobiotic compound (33). The first enzyme of the *dnt* pathway is a Rieske nonheme iron oxygenase that evolved from a precursor protein that acts on naphthalene (34). The extant catabolic pathway is still evolving, as its substrate profile and regulation have features of the antecedent route. When *Burkholderia* sp. R34 is exposed to 2,4-DNT, the substrate is indeed degraded, but cells undergo a massive intracellular production of ROS (35) stemming from the first reaction (i.e., dioxygenation of the nitroaromatic compound to yield 4-methyl-5-nitrocatechol). While ROS formation results in killing most of the microbial population, this DNA-damaging and protein-perturbing agent causes the surviving cells to diversify genetically (35). At least part of the ROS-triggered DNA mutagenesis can be traced to misincorporation of 8-hydroxy-2′-deoxyguanosine (8-oxoG) to DNA, although other mechanisms of ROS-mediated inhibition of the DNA mismatch repair system could be at play (36–38). The example of 2,4-DNT degradation illustrates how stress arising from an abortive metabolic reaction can paradoxically promote evolution of novel routes, as genetic diversification fosters exploration of the solution space by the whole bacterial population, plausibly leading to an optimized catabolic outcome that ultimately becomes fixed in the genome. One consequence is that the evolution of aerobic degradation pathways for xenoaromatic compounds can occur only in bacterial hosts able to cope with intracellular ROS generation to a level that allows genetic diversification without surpassing a deadly threshold. The most common mechanism to counter oxidative stress involves the action of detoxifying enzymes (e.g., catalases, peroxidases, and hydroperoxide reductases) that inactivate ROS (39). The corresponding reactions are ultimately fed by metabolic NADPH (40, 41), which provides the reductive currency to counteract the noxious effects of ROS, e.g., via reduced glutathione (42, 43).

In this work, we addressed the effect of the background redox metabolism on the evolvability of environmental bacteria hosting a new biodegradation/biotransformation pathway. We implanted the *dnt* route for 2,4-DNT catabolism in the genome of the model soil bacterium Pseudomonas putida and inspected the effects of metabolizing this compound on intracellular ROS production, redox stress, and genetic variability resulting from DNA mutagenesis. P. putida is a frequent host of pathways for aerobic degradation of aromatics, and it is a habitual carrier of both evolving routes and naturally optimized pathways (44–46). These qualities are generally attributed to the distinct core metabolic network of this bacterium, geared to maintain high NADPH levels (47, 48), which is further reinforced through the action of stress-induced pyridine nucleotide transhydrogenases (49). The results below indicate that ROS, resulting from faulty reactions of the *dnt* pathway on 2,4-DNT, are translated into genetic diversification of the host in a fashion that depends on its redox status—and therefore that evolvability of new traits is ultimately tuned by the background metabolism of the bacterial host. Consequently, some bacteria seem to be more suitable in hosting evolution of new pathways and delivering their activities in sustained form, compared to others in which the background metabolic network cannot cope with the massive formation of ROS.

## RESULTS

### Construction of a stable 2,4-DNT–degrading P. putida strain.

**FIG 1:**
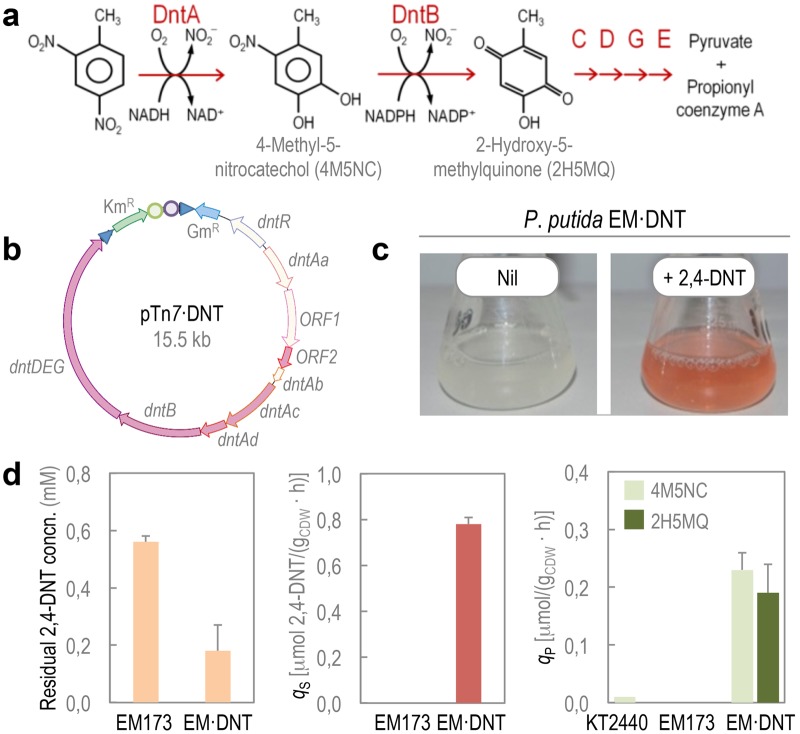
Construction and phenotypic testing of a 2,4-DNT–degrading P. putida strain. (a) 2,4-DNT degradation pathway in *Burkholderia* sp. R34. The catabolic route begins with the action of DntA, a 2,4-DNT dioxygenase belonging to the Rieske nonheme iron family that hydroxylates the aromatic ring in positions 4 and 5 to yield 4M5NC, releasing the first nitro substituent. The substituted catechol is subsequently mono-oxygenated by DntB, a 4M5NC hydroxylase that eliminates the remaining nitro group in the structure, yielding 2H5MQ. The rest of the reactions in the pathway (executed by DntCDGE) include a ring cleavage reaction and channeling of the products toward central carbon metabolism. (b) Assembly of the genes encoding proteins for the whole route for 2,4-DNT degradation, along with the *dntR*-encoded regulatory protein, in a synthetic Tn*7* transposon. The resulting plasmid, pTn*7*⋅DNT, was delivered at the defined *att*Tn*7* chromosomal site of the target host for stable insertion of the *dnt* gene cluster. (c) Qualitative testing of the recombinant P. putida EM⋅DNT strain, in which the *dnt* genes have been stably inserted in the chromosome of strain EM173. The appearance of a reddish color in cultures added with 2,4-DNT at 0.5 mM indicates presence of 2H5MQ. (d) Quantification of kinetic parameters in cultures of P. putida KT2440 (wild-type strain), EM173 (a reduced-genome derivative of strain KT2440), and EM⋅DNT (expressing the *dnt* genes) grown in the presence of 2,4-DNT at 0.5 mM. The specific rates of 2,4-DNT consumption (*q*_S_) and formation of 4M5NC and 2H5MQ (*q*_P_) were calculated by measuring the concentrations of the substrate and the products in culture supernatants. Bars represent mean values ± SD (*n* = 4) obtained after 24 h of incubation. Concn., concentration; CDW, cell dry weight.

The 2,4-DNT degradation pathway of *Burkholderia* sp. R34 converts the aromatic substrate into propionyl-coenzyme A and pyruvate through the sequential action of six enzymes (DntA to DntE)(Fig. 1a). The genes encoding the entire pathway (i.e., *dntAaAbAcAd dntB dntD dntE dntG*), along with an open reading frame (ORF) encoding a native regulatory protein (i.e., DntR) (50), were cloned by PCR from *Burkholderia* sp. R34, assembled in a synthetic Tn*7*-based vector (Fig. 1b), and delivered into the chromosome of P. putida EM173. This strain is a derivative of wild-type KT2440 devoid of four prophages and the endogenous Tn*7* transposase (51) and displays enhanced genetic stability, a trait exploited when manipulating enzymes involved in harsh biochemical reactions (52). After checking the proper insertion of the genes into the target chromosome by PCR, the capability of the resulting strain (termed P. putida EM⋅DNT) to degrade 2,4-DNT was tested. For this, cells were grown overnight at 30°C and then diluted in fresh M9 minimal medium with 0.4% (wt/vol) succinate. When cultures reached an optical density at 600 nm (OD_600_) of 0.5, 2,4-DNT was added at 0.5 mM. After 3 h of incubation—and similarly to the original 2,4-DNT–degrading *Burkholderia* strain (35)—P. putida EM⋅DNT secreted distinctively colored metabolites (Fig. 1c), which indicated the activity of the *dnt* route in the surrogate P. putida host.

As a quantitative measure of the activities implanted in P. putida, both the consumption of 2,4-DNT and the appearance of key metabolic intermediates in the route were assessed by gas chromatography coupled to mass spectrometry (Fig. 1d). After 24 h of incubation, P. putida EM⋅DNT processed 64% of the aromatic substrate, with a specific consumption rate of 0.78 ± 0.04 µmol 2,4-DNT/g cell dry weight (g_CDW_)/h. The observed rate of 2,4-DNT consumption was comparable to that previously shown in cultures of *Burkholderia* sp. R34, the original host of the pathway, in which 70% of the substrate was consumed after 24 h of incubation (8, 35). As expected, no substrate consumption was detected in cultures of the parental strain EM173. Both 4-methyl-5-nitrocatechol and 2-hydroxy-5-methylquinone, the metabolic products of DntA and DntB, respectively (53, 54), were detected in supernatants of P. putida EM⋅DNT cultures with added 2,4-DNT but not in control experiments. The specific formation rates of 4-methyl-5-nitrocatechol and 2-hydroxy-5-methylquinone in these cultures were 0.24 ± 0.03 and 0.19 ± 0.05 µmol/g_CDW_/h, respectively. The appearance of the colored species in culture supernatants indicated that at least the first two steps of the biotransformation of 2,4-DNT into the corresponding metabolic intermediates are catalytically active in the surrogate host of the degradation pathway. Taken together, these results show that the 2,4-DNT degradation route grafted into P. putida (and, in particular, the ROS-generating DntA Rieske nonheme iron oxygenase that processes 2,4-DNT) was active under the culture conditions tested.

### 2,4-DNT degradation by P. putida EM⋅DNT results in ROS generation and activation of the cellular response to oxidative stress.

**FIG 2:**
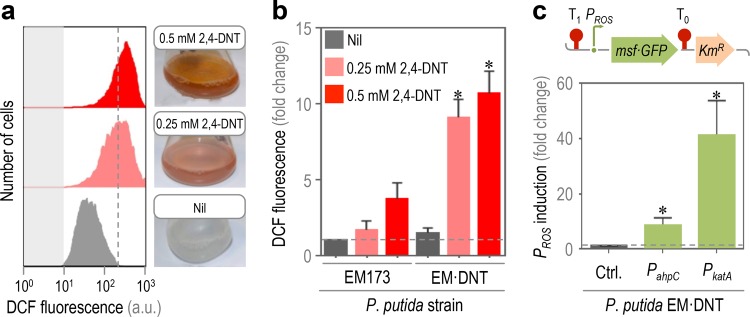
Phenotypic and transcriptional stress response of P. putida EM⋅DNT exposed to 2,4-DNT. (a and b) Flow cytometry-assisted determination of ROS formation. (a) Histograms of raw data from untreated cells (Nil, added with DMSO, the 2,4-DNT solvent carrier) and cells exposed to 2,4-DNT at 0.25 or 0.5 mM. Cell suspensions were treated with the ROS-sensitive probe H_2_DCF-DA, and the resulting dichlorofluorescein (DCF) fluorescence levels were recorded in at least 15,000 individual cells. Gray rectangles indicate the maximum fluorescence in cells without addition of H_2_DCF-DA. A representative experiment per condition is shown in the diagram. (b) Fold change of the geometric mean (*x* mean) of DCF fluorescence levels for each experimental condition in P. putida EM173 (parental strain) and EM⋅DNT (carries the *dnt* gene cluster). Bars represent the average of *x* means ± SD (*n* = 6), and the asterisk identifies significant differences at a *P* level of <0.05 as determined indicated by the Mann-Whitney U test (comparing DCF fluorescence levels in cells exposed to 2,4-DNT with that in nontreated cultures). The dashed gray line indicates the basal level of DCF fluorescence in the control experiment for each strain. (c) Transcriptional activities of stress-responsive promoters. Two different oxidative stress reporters were constructed by placing the corresponding promoter (*P*_*ROS*_) in a pBBR1-based, kanamycin-resistant (Km^r^) vector bearing the promoterless gene encoding the monomeric and superfolder GFP (msf⋅GFP). The *P*_*ROS*_ → *msf*⋅*GFP* construct was transcriptionally insulated by means of the T0 and T1 terminators. Elements are not drawn to scale. The *x* mean of the msf⋅GFP fluorescence was detected by flow cytometry in P. putida EM⋅DNT exposed to 2,4-DNT at 0.5 mM. The resulting msf⋅GFP fluorescence was compared to that in cells harvested from cultures that were not treated with 2,4-DNT (Ctrl., baseline indicated with a dashed gray line). Bars represent the mean values of the *x* means of the msf⋅GFP fluorescence ± SD (*n* = 4), and the asterisks identify significant differences at a *P* level of <0.05 as determined using Student’s *t* test.

Despite having the necessary genetic and biochemical complement, *Burkholderia* sp. R34 grows poorly on 2,4-DNT as the sole carbon source. This difficulty that can be traced to the formation of ROS upon exposure of the cells to the substrate of the biodegradation pathway (35). Against this background, we evaluated the generation of ROS in P. putida EM⋅DNT when exposed to 2,4-DNT at either 0.25 or 0.5 mM by assessing the fluorescence brought about by the ROS-sensitive dye 2′,7′-dichlorodihydrofluorescein diacetate (H_2_DCF-DA) in single cells by using flow cytometry (Fig. 2a). The amount of ROS proportionally increased with respect to the concentration of 2,4-DNT, indicating that substrate consumption generates oxidative stress. The proportionality between the activity of the degradation pathway and ROS generation was qualitatively assessed in these experiments, since the amount of 2-hydroxy-5-methylquinone produced by the cells increased with the concentration of 2,4-DNT in the same fashion as ROS formation did. Quantification of ROS indicated that the mere exposure of the cells to 2,4-DNT is not the main cause of ROS generation; instead, the biochemical transformation of the substrate leads to endogenous oxidative stress. Indeed, P. putida EM⋅DNT had a ca. 10-fold increase in ROS formation upon exposure to 2,4-DNT (Fig. 2b). In contrast, strain EM173 had a lower accumulation of ROS (less than 4-fold increase) than strain EM⋅DNT when challenged with 2,4-DNT, even at the highest substrate concentration tested. While biotransformation intermediates 4-methyl-5-nitrocatechol and 2-hydroxy-5-methylquinone (55) could be mutagenic, we previously demonstrated that increasing turnover of metabolites downstream of 4-methyl-5-nitrocatechol by controlled overexpression of *dntB* in *Burkholderia* sp. R34 does not result in a decrease of ROS formation (35). This indicates that the activity of the first enzyme of the pathway (and not the metabolic intermediates therein) is responsible for the bulk of the oxidative stress observed upon addition of 2,4-DNT.

Yet, is the observed ROS formation connected to the activation of stress responses in the engineered P. putida strain? To answer this question, the transcriptional activities of genes involved in the oxidative stress response (i.e., *ahpC* [PP_2439, alkyl hydroperoxide reductase] and *katA* [PP_0481, a catalase]) were studied by fusing the corresponding promoter region of these two genes to the reporter monomeric superfolder green fluorescent protein (msf⋅GFP) (Fig. 2c). The ROS reporter plasmids or pSEVA237M, the promoterless, msf⋅GFP-containing vector (56), were individually transformed into P. putida EM⋅DNT, and the msf·GFP signal was evaluated in cultures exposed to 2,4-DNT by using flow cytometry. The output signal qualitatively followed the same trend as observed for ROS accumulation (Fig. 2b): the induction of the two oxidative stress-responsive promoters significantly increased in the presence of the aromatic compound (i.e., 10- and 42-fold increase in the transcriptional activities of *P*_*ahpC*_ and *P*_*katA*_, respectively, when cells were exposed to 2,4-DNT at 0.25 mM). In accordance with previous observations in *Burkholderia* sp. R34 (35), these results indicated that cells carrying the enzymes that are needed to process 2,4-DNT undergo oxidative stress conditions upon exposure to the substrate of the degradation pathway.

### Assessment of effects of 2,4-DNT degradation on the SOS response and recombinogenic activity.

**FIG 3:**
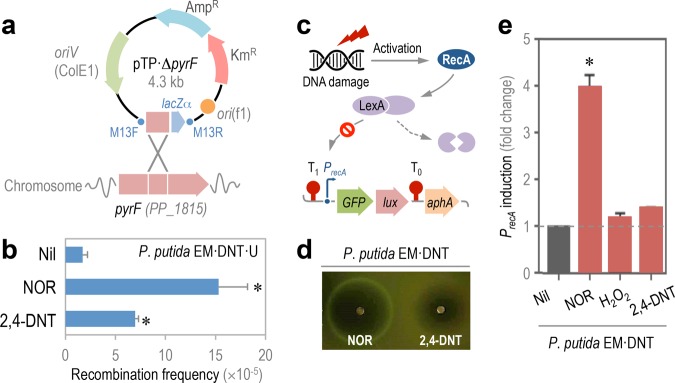
Effect of 2,4-DNT degradation on DNA recombination and SOS response. (a) Construction of a reporter P. putida strain to study DNA recombination. A suicide, integrative plasmid, obtained as detailed in Text S1 in the supplemental material, was used to disrupt the *pyrF* gene of P. putida, which results in uracil auxotrophy. The frequency of excision of the plasmid from the chromosome of P. putida EM⋅DNT⋅U (i.e., reversion to prototrophy) was adopted as an indication of DNA recombination. (b) DNA recombination frequency upon exposure to 2,4-DNT or norfloxacin (NOR). Bars represent mean values ± SD (*n* = 4), and the asterisks identify significant differences at a *P* level of <0.05, determined using Student’s *t* test. (c) General mechanism of SOS response upon DNA damage. The RecA protein, stimulated by either damaged or single-stranded DNA, triggers the inactivation of LexA (a repressor of the SOS response genes), thereby inducing the response. The LexA degradation-dependent activation of the *recA* promoter of P. putida was used as a proxy of the SOS response by constructing an msf⋅GFP-based biosensor. Elements in the outline are not drawn to scale. (d) Testing of the SOS response biosensor in soft agar experiments. P. putida EM⋅DNT was transformed with the reporter plasmid, and two filter paper disks, soaked with either NOR or 2,4-DNT, were applied onto the bacterial lawn. The plates were photographed under blue light after 24 h of incubation. (e) Quantification of the SOS response biosensor activity in cultures of P. putida EM⋅DNT in the presence of the additives indicated. Bars represent the mean values of the fold change in msf⋅GFP fluorescence ± SD (*n* = 4), and the asterisks identify significant differences at a *P* level of <0.05 as determined using Student’s *t* test. The baseline in cultures with no additives (Nil) is indicated with a dashed gray line.

Since the hypothesis underlying this work is that endogenous oxidative stress brought about by the biotransformation of an aromatic substrate could result in genetic novelty, we explored two types of mutagenic effects on the genome of P. putida. In the first place, and in order to evaluate if the degradation of 2,4-DNT promotes *recA*-mediated homologous DNA recombination, a reporter strain was designed as follows. An internal region of the *pyrF* gene of P. putida (PP_1815, orotidine 5′-phosphate decarboxylase) was cloned into a vector that cannot replicate in *Pseudomonas* species (Fig. 3a). Integration of the entire pTP⋅Δ*pyrF* plasmid in the target locus of strain EM173 blocked the PyrF activity altogether. Since this enzyme catalyzes the last essential step in the *de novo* biosynthesis of pyrimidines (57), the resulting P. putida insertion mutant is rendered auxotroph for uracil (Ura). The extent of DNA recombination brought about by selected factors can be assessed in this strain by scoring the reversion to prototrophy due to excision of the plasmid DNA inserted in the *pyrF* locus. The 2,4-DNT degradation pathway was integrated in the chromosome of this reporter strain as previously indicated (58), giving rise to P. putida EM⋅DNT⋅U (i.e., EM173 *dntABDEG dntR* Δ*pyrF*, Ura^–^). P. putida EM⋅DNT⋅U was challenged with 2,4-DNT at 0.5 mM, and cells were plated onto M9 minimal medium with or without Ura. The frequency of appearance of P. putida prototroph (Ura^+^) clones was quantified (Fig. 3b), and the strain degrading 2,4-DNT showed a 4-fold increase in the frequency of recombination compared to control conditions. Norfloxacin (NOR) was added in cultures used as positive controls. NOR is a fluoroquinolone that interferes with the maintenance of chromosomal topology by targeting DNA gyrase and topoisomerase IV, trapping these enzymes at the DNA cleavage stage and thereby preventing strand rejoining (59). Introduction of double-stranded DNA breaks following topoisomerase inhibition by NOR thus induces the SOS response (38, 60). In the presence of NOR, we detected a 7-fold increase in DNA recombination using our reporter system (Fig. 3b).

Since degradation of 2,4-DNT brings about an increase in the frequency of DNA recombination, we wondered whether this would result in the activation of the SOS response (i.e., DNA damage response), which is stimulated by single- or double-stranded breaks in genomic DNA (61). When DNA is injured, the RecA protein binds to DNA in the damaged region to form a filament. This filament interacts with a dimer of the LexA transcriptional repressor, activating its self-cleavage and causing the dissociation of LexA from its targets and inducing the SOS regulon (Fig. 3c). The promoter of *recA* is one of the targets of LexA (62), an occurrence that was exploited in this work by constructing a biosensor of RecA activity. The promoter region of *recA* (PP_1629), including the putative LexA binding site, was cloned in front of a promoterless *GFP*-*luxCDABE* dual reporter system (63). The resulting reporter plasmid was introduced into P. putida EM⋅DNT, and the system was tested by exposing the cells to NOR and 2,4-DNT in a qualitative soft agar diffusion test (Fig. 3d). Exposure of strain EM⋅DNT carrying the RecA reporter to NOR resulted in a distinct halo of growth inhibition, the boundaries of which gave off a strong GFP signal when observed under UV light. 2,4-DNT, in contrast, did not seem to elicit a similar response in the cells carrying the RecA reporter. A similar pattern was observed in GFP-dependent fluorescence when the experiment was repeated in liquid cultures of P. putida EM⋅DNT bearing the SOS response biosensor, as quantified by flow cytometry (Fig. 3d). Addition to NOR at subinhibitory concentrations (250 ng ml^−1^) caused a 4-fold induction of the RecA reporter, whereas neither 2,4-DNT nor H_2_O_2_ resulted in any significant activation of the SOS response. Taken together, these results indicate that degradation of the aromatic substrate promotes DNA recombination extent, without significantly affecting the activity of the SOS response. The next question was whether other forms of mutagenesis, such as point mutations in genomic DNA, could be also correlated to 2,4-DNT biotransformation by the engineered P. putida strain.

### Biotransformation of 2,4-DNT in P. putida barely affects DNA mutagenesis.

Although ROS production stemming from 2,4-DNT metabolism in P. putida EM⋅DNT does not trigger a significant SOS response and only stimulates recombination to a moderate degree, it was still possible that oxidative damage to DNA, generation of 8-oxoG, and general stress-induced relaxation of mismatch repair could increase the overall level of DNA mutagenesis. To inspect this possibility (which was observed in *Burkholderia* sp. R34) the mutagenesis rate was assessed by exposing P. putida EM⋅DNT cells to 2,4-DNT and counting the number of rifampin-resistant (Rif^r^) colonies after plating on a solid culture medium. The antibacterial effects of Rif are based on its ability to bind the β-subunit of the RNA polymerase, thereby blocking the elongation of the nascent RNA molecule. Rif^r^ clones usually harbor RpoB mutations in amino acid residues that make contact with Rif (64), rendering RNA polymerase insensitive to the antibiotic (65, 66). Under our experimental conditions, the background rate (*M*) of Rif^r^ clones (i.e., P. putida EM⋅DNT cells incubated in the presence of dimethyl sulfoxide [DMSO]), was (9.1 ± 0.7) × 10^−9^. Exposure of P. putida EM⋅DNT cells to 2,4-DNT increased this level of mutagenesis by a mere 28% (and the difference from control experiments was not statistically significant). This was an unexpected occurrence, as high levels of endogenous ROS are translated onto a mutagenic state in other bacteria. That this was not the case in the strain constructed in this study indicated that the molecular mechanisms that connect ROS stress with DNA mutagenesis in P. putida are plausibly different from those operating in other bacterial species that other factors could be at play. Yet, what could such key factors be? One metabolic signature that characterizes P. putida is its highly reductive redox metabolism. We thus wondered next whether the interplay between ROS production and DNA mutagenesis could be shielded by such a background metabolism that is so characteristic of this species.

### Altering the redox status of P. putida increases the mutagenic effect of the 2,4-DNT degradation pathway.

**FIG 4:**
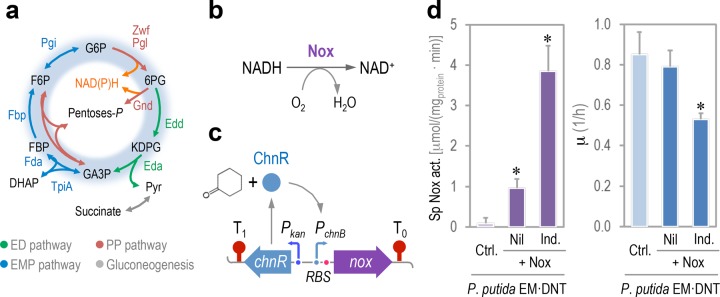
Perturbation of the redox metabolism of P. putida. (a) Simplified scheme of the upper carbon metabolism of P. putida. Note that redox balance is maintained through the action of the EDEMP cycle, indicated in blue shading in the diagram. Abbreviations: ED pathway, Entner-Doudoroff pathway; EMP pathway: Embden-Meyerhof-Parnas pathway; PP pathway, pentose phosphate pathway; G6P, glucose-6-P; F6P, fructose-6-P; FBP, fructose-1,6-P_2_; DHAP, dihydroxyacetone-P; GA3P, glyceraldehyde-3-P; 6PG, 6-phosphogluconate; KDPG, 2-keto-3-deoxy-6-phosphogluconate. (b) Reaction catalyzed by the water-forming Nox NADH oxidase from Streptococcus pneumoniae. (c) Construction of a synthetic NADH-burning device for tightly regulated expression of *nox*. The gene encoding Nox was placed under the control of the *P*_*chnB*_ promoter, which responds to the cyclohexanone-activated ChnR regulator. Elements in the outline are not drawn to scale. (d) Nox activity and impact of endogenous redox imbalance on the overall physiology of P. putida EM⋅DNT. The specific (Sp) *in vitro* Nox activity was compared in P. putida EM⋅DNT carrying either the empty pSEVA2311 vector (Ctrl.) or plasmid pS2311⋅Nox, with (Ind.) or without (Nil) addition of cyclohexanone at 0.1 mM to induce the expression of *nox*. The specific growth rate (μ) was determined in the same cultures. Each bar represents the mean value of the corresponding parameter ± SD (*n* = 5), and the asterisks identify significant differences at a *P* level of <0.05 as determined using Student’s *t* test.

P. putida maintains an adequate supply of reducing power through the activity of the EDEMP cycle (47), a core metabolic architecture that combines individual biochemical steps from the Entner-Doudoroff, the Embden-Meyerhof-Parnas, and the pentose phosphate pathways (Fig. 4a). By recycling part of the pool of triose-phosphate back to hexose phosphates, this metabolic cycle mediates NAD(P)H formation and enables catabolic overproduction of reduced pyridine nucleotides. This evolutionary-driven metabolic occurrence helps explain the very high resistance to environmental insults (e.g., oxidative stress) displayed by P. putida (67–69). Moreover, antioxidant responses in bacteria exposed to xenobiotics rely on the generation of the reducing power that the cells use to counteract ROS formation. For instance, AhpC, a hydroperoxide-detoxifying enzyme (Fig. 2c), is reduced by peroxiredoxin reductase (AhpF), a process that requires NADH (70). On this basis, and taking into consideration that (i) 2,4-DNT degradation results in the generation of ROS and (ii) the oxidative damage caused by ROS stimulates DNA damage, we set to explore the relationship between redox status and DNA mutagenesis in P. putida EM⋅DNT. To investigate this issue, the redox status of P. putida EM⋅DNT was artificially perturbed by altering the intracellular availability of reduced nicotinamide cofactors through conditional expression of the *nox* gene of Streptococcus pneumoniae (71, 72). Nox is a NADH-specific oxidase enzyme from that converts O_2_ to H_2_O with negligible formation of H_2_O_2_ (Fig. 4b). Cellular energy demands are not affected by Nox, and thus *nox* expression allows for the specific investigation of the impact of NADH oxidation without affecting the overall fitness of the cells (73). A synthetic metabolic module was designed for this purpose, in which the gene encoding NADH oxidase from S. pneumoniae was placed under control of the orthogonal ChnR/*P*_*chnB*_ expression system (Fig. 4c), which is inducible by cyclohexanone (74, 75). We tested the effect of such an NADH-burning device on the overall physiology of P. putida EM⋅DNT by measuring the specific Nox activity in cell-free extracts and evaluating growth rates on succinate cultures (Fig. 4d). Induction of the synthetic device by addition of 0.1 mM cyclohexanone resulted in a specific Nox activity of 3.9 ± 0.6 µmol/mg of protein/min, ca. 40-fold higher than the background oxidase activity in the cell extract of the control strain transformed with the empty vector. Under these conditions, the specific growth rate of P. putida EM⋅DNT was reduced by ca. 30%. As a direct indication of the role of Nox in mediating a redox imbalance, the reduced nucleotide content was evaluated in cell-free extracts of P. putida EM⋅DNT. The NADH concentration was 176 ± 25 and 103 ± 38 µM for the strain carrying the empty pSEVA2311 vector or plasmid pS2311⋅Nox, respectively, demonstrating that the overexpression of *nox* in P. putida cells decreases the reducing power content. The next step was to evaluate if an alteration in the NADH content affects DNA mutagenesis.

**FIG 5:**
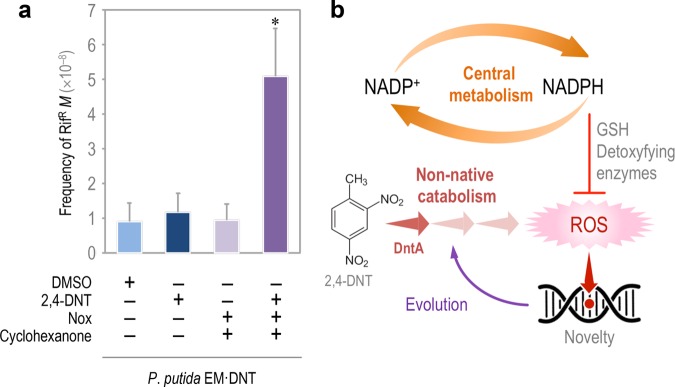
DNA mutagenesis in redox-challenged P. putida EM⋅DNT cells exposed to 2,4-DNT. (a) Frequency of spontaneous rifampin-resistant mutants (*M*) in cells exposed to 2,4-DNT at 0.5 mM, DMSO (the 2,4-DNT solvent carrier), cyclohexanone at 0.1 mM (inducer of the synthetic device driving the expression of the Nox NADH oxidase), or combinations thereof. The dimensionless frequencies of mutation were calculated using the Ma-Sandri-Sarkar maximum likelihood estimator. Bars represent the mean *M* values ± SD (*n* = 4), and the asterisks identify significant differences at a *P* level of <0.05 as determined with Student’s *t* test. (b) Model for metabolism-driven evolution. Faulty redox reactions on novel substrates trigger ROS, which may cause direct or indirect damage to DNA in a fashion dependent on the background metabolism, which provides the reducing power necessary to fuel detoxifying enzymes (typically dependent on reduced glutathione [GSH]).

P. putida EM⋅DNT was transformed with the *nox*-expressing plasmid or the empty pSEVA2311 vector, and the resulting strains were exposed to different combinations of 2,4-DNT and cyclohexanone. The rate of mutagenesis, *M*, was explored by assessing the appearance of Rif^r^ clones after each treatment (Fig. 5a). As shown in the figure, the exposure of the cells to 2,4-DNT or activation of Nox individually did not result in a significant increase in DNA mutagenesis. The combination of the two treatments, however, produced a 6-fold increase in *M*, indicating that the redox imbalance introduced by Nox exacerbated the mutagenic impact of 2,4-DNT. Yet, what is the nature of the mutations that lead to Rif^r^ in such a redox-stressed scenario?

### The spectrum of *rpoB* mutations depends on the intracellular redox status.

Mutations in *rpoB* that confer Rif^r^ are well conserved among prokaryotes, and the amino acid changes that produce this phenotype can be grouped in three clusters in a central region of RpoB (65). We sequenced ca. 100 clones per experimental condition to obtain the spectrum of mutations in each case (Table 1). All the mutations detected in this analysis were located in cluster I of RpoB (between nucleotides 1,528 and 1,626 of *rpoB*, as defined by Jatsenko et al. [65]), and they could be grouped into eight categories. Five of the categories were transitions (i.e., A → G and C → T), two of them were transversions (i.e., A → T and C → A), and the rest of the *rpoB* mutations found in Rif^r^ clones were grouped as “other” (a category that included the less abundant *rpoB* mutations). The two predominant mutations found resulted in Q518L and D521G changes in RpoB, and their frequency of appearance did not suffer any significant change across the experimental treatments tested here. Interestingly, the frequency of C → A transversion that generates an L538I change in RpoB was influenced by both exposure to 2,4-DNT (2-fold increase) and the activation of Nox (4-fold increase). The effect of the two treatments was additive, further linking biotransformation of the substrate and redox status of the cells with DNA mutagenesis. C → A transversions are known to be caused by the presence of 8-oxoG in the DNA (76), which results from the attack of ROS on purine residues. This result thus indicated that oxidative stress caused by 2,4-DNT degradation results in oxidation of purines (as observed in *Burkholderia* sp. R34), but it also shows that the more reductive redox status of P. putida provides effective protection against this mutagenic effect. Other mutations arising by exposing the cells to 2,4-DNT had a less clear origin, e.g., the activity of the widely distributed Y-family of DNA polymerases (77). Members of this family are characterized not only by their ability to replicate damaged DNA but also by their lack of processivity and low fidelity when copying undamaged template. In P. putida KT2440, this protein category is represented by DinB (PP_1203) and the error-prone DNA polymerase DnaEB (PP_3119) (78). In sum, the spectrum of *rpoB* mutations detected in our study suggests that ROS-induced mutagenesis triggered by 2,4-DNT metabolism merges direct damage to DNA caused by misincorporation of 8-oxoG with other stress response mechanisms that relax fidelity of DNA replication and/or prevent repair.

**TABLE 1:** Nature and frequency of mutations in *rpoB* (PP_0447) conferring resistance to rifampin^a^

Mutation	P. putida EM⋅DNT carrying:					
	pSEVA2311 (empty vector)			pS2311⋅Nox (NADH oxidase)		
	DMSO	2,4-DNT	2,4-DNT + CHX	DMSO	2,4-DNT	2,4-DNT + CHX
Q518L (A → T)	31	27	29	32	19	15
Q518R (A → G)	14	11	13	11	11	10
D521G (A →G)	21	25	22	19	26	24
H531R (A → G)	12	7	5	15	12	6
H531Y (C → T)	8	9	10	7	4	8
S536F (C → T)	9	4	3	10	8	9
L538I (C → A)	1	11	13	5	11	21
Other	4	6	5	1	9	7

a The percentage of amino acid changes in RpoB is indicated along with the DNA mutation from which the substitution originated. These percentages were calculated by sequencing an internal region of *rpoB* in at least 90 rifampin-resistant clones under each experimental condition. The mutations indicated in the list were selected since they were observed across all the experimental conditions tested. The remaining point mutations are grouped as “other.” DMSO, dimethyl sulfoxide; 2,4-DNT, 2,4-dinitrotoluene; CHX, cyclohexanone.

## DISCUSSION

**FIG 6:**
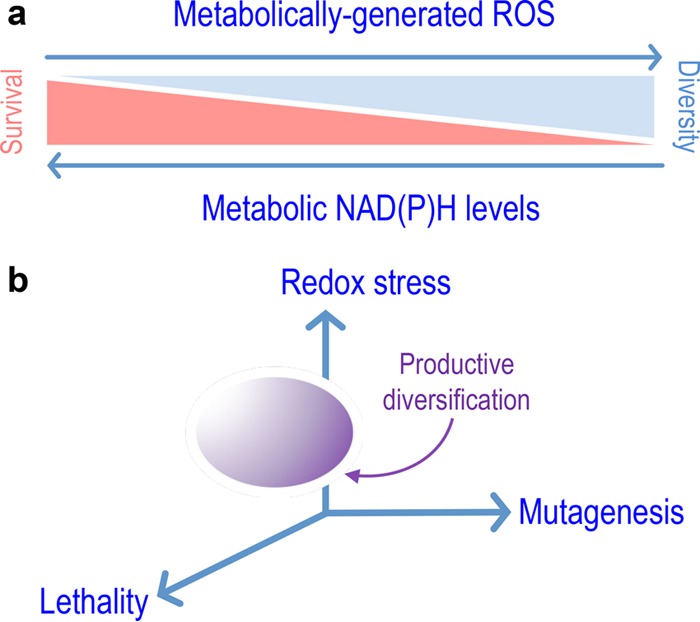
Evolutionary hormesis driven by metabolic formation of reactive oxygen species. (a) Interplay between redox stress and genetic and functional diversification of bacteria. As explained in the text, the action of ROS as a direct or indirect DNA mutagenesis agent is checked (and kept at bay) by the endogenous levels of reductive currency [i.e., availability of NAD(P)H]. This situation defines a scenario in which the gain of diversity occurs at the expense of population survival. (b) Species-specific definition of a productive diversification space. We argue that the evolvability of given bacterial species and the potential to host novel catabolic pathways is framed by the ability of bacteria to endure ROS-driven stress in a fashion that depends on their particular redox metabolism. These independent elements demarcate a space of productive diversification, i.e., an optimum in the novelty supply rate of the system (110), which fosters the exploration of the evolutionary solution space and which could be unique for each bacterial species.

Evolvability of bacteria for conquering novel nutritional and physicochemical landscapes is the result of the interplay between endogenous and exogenous factors. Propagation of new pathways through a bacterial population is fostered by different mechanisms of horizontal gene transfer, e.g., conjugation, transformation, and phage-mediated or ICE (integrative and conjugative elements)-mediated DNA acquisition (79, 80). In this case, the emergence of genetic and functional diversity largely relies on the balance between the effectiveness of the transfer events and the ability of the recipient bacteria to gain an advantage thereof. Not surprisingly, microbial species with a high capacity to receive foreign DNA usually evolve new traits (81)—sometimes as undesirable as antibiotic resistances (6). However, horizontal gene transfer does not account for the onset of fresh routes for xenobiotic catabolism, which require mutation of preexisting pathways to reshape their substrate range and rewire and connect their biochemical outputs to the existing metabolic network in the host. Since these changes must necessarily occur in single bacteria, one can safely predict that the more highly mutagenic regime they display, the faster the solution space will be explored―and an optimum will be eventually found. Two common triggers of such mutagenic regimes include carbon starvation and endogenous production of ROS (82–85). Given that these mechanisms are virtually universal through all aerobic bacteria, the question arises why most aerobic routes for degradation of xenobiotic aromatics involve the action of Rieske nonheme iron oxygenases borne by pseudomonads and closely related bacteria. The results described in this article provide some rationale for this longstanding observation. The mechanism of this type of oxygenases at their reaction center involves activation of an oxygen molecule by an Fe-S cluster to make the oxygen reactive toward the aromatic substrate (86, 87). Even with a good enzyme-substrate fit, purely stochastic effects at the atomic level may result in coupling failure and release of ROS, the discharge of which will increase if the fitting of the substrate to the enzyme is worse (24–27). We have argued that, because of its mutagenic potential, the release of ROS is a major driver of the evolution of the cognate bacterial population toward a new biodegradative target (35). Yet, why are such biodegradative abilities so conspicuous in pseudomonads? The data above suggest that, while ROS production during 2,4-DNT degradation is unavoidable, DNA mutagenesis is ultimately controlled by the endogenous redox status of the corresponding cells. The highly reductive NAD(P)H regime associated with the distinct central carbon metabolism of P. putida thus seems to render this species well suited for hosting pathways involving strong redox transactions (Fig. 5b and 6a). At the same time, a dearth in NAD(P)H availability would result in a high DNA mutagenesis regime. Although data supporting this notion have been generated in only two environmental bacteria (Pseudomonas putida and *Burkholderia* spp.), it is likely that they are specific cases of a general evolutionary principle that is reminiscent of the concept of antifragility (35, 88) or hormesis (89, 90), i.e., beneficial effects of a low dose of a toxic input (91, 92), observed in other biological systems. Interestingly, hormetic dose-response mechanisms have been put forward to explain the survival of bacteria exposed to different types of antibiotics (93–95). Under this framework, there seems to be an optimum in the diversity-generating regime caused by stress (e.g., ROS) that is enough to generate sufficient evolutionary bet-hedging for coping with a new nutrient or physicochemical condition while keeping the thereby diversified population—but also damaged population—within viable levels (Fig. 6b). It is within this solution space that bacterial cells equipped with novel catabolic pathways for new substrates are to be found. The data above suggest that such a window of productive innovation is shaped by the background metabolic network, specifically by the redox regime. ROS formation has been observed in other environmental bacteria that employ biodegradation pathways for xenobiotics (96–99), and it is plausible that similar evolutionary processes apply in other instances.

That the redox metabolic status (either inherent or transient) controls evolvability of selectable traits in bacteria raises some corollaries worth follow-up. For instance, ROS may accelerate the emergence of new antibiotic resistances in species subject to endogenous oxidative stress but endowed with a weaker redox metabolism. This opens the possibility of targeting redox biochemistry of virulent strains with drugs that perturb the NAD(P)^+^/NAD(P)H balance. Moreover, our data suggest that some members of a given microbial community could be more innovative than others on the basis of their innate redox metabolism. There could be innovation specialists that nurture genetic diversification and pass the results to less mutable members of a multispecies bacterial consortium through horizontal gene transfer. Finally, ROS-mediated genetic innovation could be repurposed for the sake of generating new pathways through directed evolution or for the optimization of biocatalytic routes for metabolic engineering (100). In any case, the connection between metabolism and evolvability should not be ignored when addressing the emergence of new traits whether for fundamental understanding of evolutionary processes or for designing new whole-cell biocatalysts.

## MATERIALS AND METHODS

### Bacterial strains and culture conditions.

Bacterial strains are listed in Table S1 in the supplemental material. *Burkholderia* sp. R34 has been previously described as a 2,4-DNT-degrading specimen (33). P. putida EM173 was used as the host of the *dnt* pathway (51). E. coli strains DH5α, CC118, CC118λ*pir*, and HB101 were employed for DNA cloning procedures as indicated in Text S1 in the supplemental material. Bacteria were grown in rich LB medium or in M9 minimal medium with sodium succinate added at 0.4% (wt/vol) with rotary agitation at 170 rpm (101). P. putida was grown at 30°C, while E. coli was grown at 37°C. When required, 2,4-DNT was added at 0.25 or 0.5 mM from a 0.5 M stock solution freshly prepared in DMSO. Control experiments included an equivalent volume of DMSO. Overnight-grown P. putida in M9 minimal medium and succinate was used as inoculum by diluting the bacterial suspension to a starting OD_600_ of 0.05. Cells were grown in the same culture medium until the OD_600_ reached 0.5, at which point the cell suspension was split: one culture served as a control, and the other one was challenged with 2,4-DNT. Other additives were as indicated, and samples were periodically taken as specified. Additives were added at the following final concentrations: ampicillin, 150 µg ml^−1^ for E. coli or 500 µg ml^−1^ for P. putida; chloramphenicol, 30 µg ml^−1^; kanamycin, 50 µg ml^−1^; streptomycin, 80 µg ml^−1^; gentamicin, 10 µg ml^−1^; uracil, 20 µg ml^−1^; H_2_O_2_, 1.5 mM; cyclohexanone, 0.1 mM. Norfloxacin was used at 25 ng ml^−1^ for stimulation of DNA recombination and at 250 ng ml^−1^ for reporter experiments. All solid media contained 15 g liter^−1^ agar.

10.1128/mBio.01512-18.2 TABLE S1  Bacterial strains and plasmids used in this study. Download TABLE S1, PDF file, 0.2 MB. Copyright © 2018 Akkaya et al. 2018 Akkaya et al. This content is distributed under the terms of the Creative Commons Attribution 4.0 International license.

10.1128/mBio.01512-18.1 TEXT S1  Supplemental materials and methods. Download TEXT S1, PDF file, 0.2 MB. Copyright © 2018 Akkaya et al. 2018 Akkaya et al. This content is distributed under the terms of the Creative Commons Attribution 4.0 International license.

### General DNA techniques and construction of recombinant P. putida strains.

DNA manipulations followed routine laboratory techniques (101). Details on the design and construction of bacterial strains and plasmids can be found in Text S1 in the supplemental material. Plasmids were introduced into P. putida strains by electroporation (102, 103).

### Determination of mutation frequencies and detection of *rpoB* mutations.

P. putida cultures were grown overnight with the appropriate antibiotics, diluted 1:10,000 into 50 ml of fresh culture medium (initial OD_600_ of 0.05) in 250-ml Erlenmeyer flasks, and incubated for 4 h. These cultures were diluted 1:4 into fresh medium (1 ml in 10-ml test tubes) and incubated for 16 h in the presence of relevant additives. Appropriate dilutions were spread on LB medium plates containing 50 µg ml^−1^ Rif. Colonies were counted after 48 h at 30°C. Luria-Delbrück fluctuation analysis was performed to study the impact of additives on the mutation rate *M*. *M* values were computed using the Ma-Sandri-Sarkar maximum likelihood estimator (ratio between the number of Rif^r^ colonies and the total viable count, corrected by plating efficiency) from at least six independent experiments per condition (104, 105). The statistical significance of mean *M* values across experiments was evaluated with Student’s *t* test. To identify the nature and frequency of point mutations in *rpoB*, ca. 100 Rif^r^ colonies were randomly collected from at least four independent experiments, and the locus was sequenced with primers indicated in the supplemental material. The BioEdit software (BitSize Bio) was used for sequence comparison.

### Analytic procedures.

The ROS-sensitive green fluorescent dye H_2_DCF-DA (Sigma-Aldrich) was used to quantitate ROS formation. Flow cytometry, preparation of cell-free extracts, metabolite extraction, and enzymatic assays followed protocols from our laboratory (106–109), and further details can be found in Text S1 in the supplemental material.

### Statistical analysis.

All reported experiments were independently repeated at least three times (as indicated in the figure legends), and mean values of the corresponding parameter and standard deviation (SD) are presented. The significance of differences when comparing results was evaluated via use of Student’s *t* test.
