# Supplementary material for: The Metabolic Redox Regime of Pseudomonas putida Tunes Its Evolvability toward Novel Xenobiotic Substrates
Source: mBio. 2018 Aug 28;9(4):e01512-18. doi: 10.1128/mBio.01512-18 (PMC6113623; doi:10.1128/mBio.01512-18)
Supplement: TABLE S1 [file mbo004184023st1.pdf]

Table S1. Bacterial strains and plasmids used in this study[a].

Bacterial strain	Relevant characteristics	Reference
Burkholderia		
R34	Wild-type strain; 2,4-DNT-degrading isolate carrying megaplasmids pJS311 and pJS312	(1)
E. coli		
DH5α	Cloning host; *supE44* Δ*lacU169*(Φ*80 lacZ*Δ*M15*) *hsdR17*(r_k^- m_k^+) *recA1 endA1 thi1 gyrA relA*	(2)
CC118	Cloning host; F⁻ Δ(*ara-leu*)*7697 araD139* Δ(*lac*)*X74 phoA*Δ*20 galEK thi rpsE rpoB*	(3)
CC118λ*pir*	Cloning host; F⁻ Δ(*ara-leu*)*7697 araD139* Δ(*lac*)*X74 phoA*Δ*20 galEK thi rpsE rpoB* λ*pir* lysogen	(4)
HB101	Helper strain; *hsdR17*(r_k^- m_k^+) *pro leu thi recA1*	(2)
P. putida		
KT2440	Wild-type strain; derivative of strain mt-2 cured of the TOL plasmid pWW0	(5)
EM173	Derivative of strain KT2440; Δprophages ΔTn7	(6)
EM·DNT	Derivative of strain EM173 carrying the complete *dnt* pathway as a Tn7 chromosomal insertion; Gm^R	This study
EM·DNT·U	Derivative of strain EM·DNT bearing a chromosomal insertion of plasmid pTP·Δ*pyrF*, uracil auxotroph; Gm^R Km^R	This study
Plasmids		
pRK600	Cm^R; *oriV*(ColE1), RK2(*mob*⁺ *tra*⁺); helper plasmid	(4)
pTNS1	Amp^R; *oriV*(R6K), *tnsABDC*; helper plasmid	(7)
pTn*7*_MCS	Km^R, Gm^R; *oriV*(R6K), *oriT*; Tn7 vector	(8)
pTn*7*·DNT	Km^R, Gm^R; *oriV*(R6K), *oriT*; derivative of vector pTn7_MCS with the *dnt* gene cluster (*dntA*$_{abcd}$*BDEG dntR*) from *Burkholderia* sp. R34	This study
pTrc99A-*nox*	Amp^R; derivative of vector pTrc99A (9) containing the *nox* gene from *Streptococcus pneumoniae* under control of $LacI^Q/P_{trc}$	(10)
pSEVA2311	Km^R; *oriV*(pBBR1), *oriT*; standard broad-host-range vector containing the ChnR/P_{chnB} expression system	(11)
pS2311·Nox	Km^R; derivative of vector pSEVA2311 with the *nox* gene from *S. pneumoniae*	This study
pGLR2	Km^R, *oriV*(RK2), *oriT*; dual *GFP-luxCDABE* reporter system	(12)
pGR·*recA*	Km^R; P_{recA} promoter fragment cloned into vector pGLR2	This study
pSEVA237M	Km^R; *oriV*(pBBR1), *oriT*; promoter-less *msf·GFP*	(13)
pSR·*ahpC*	Km^R; P_{ahpC} promoter fragment cloned into vector pSEVA237M	(14, 15)
pSR·*katA*	Km^R; P_{katA} promoter fragment cloned into vector pSEVA237M	(14, 15)
pCR2.1-TOPO	Amp^R, Km^R; standard cloning vector for *Taq* DNA polymerase-amplified PCR products	Invitrogen
pTP·Δ*pyrF*	Amp^R, Km^R; derivative of vector pCR2.1-TOPO bearing a 500-bp internal region of the *pyrF* gene from strain KT2440	This study

[a] The abbreviations used in this table are as follows: Amp, ampicillin; Km, kanamycin; Gm, gentamycin; and Cm, chloramphenicol.

REFERENCES

1. **Nishino SF, Paoli GC, Spain JC.** 2000. Aerobic degradation of dinitrotoluenes and pathway for bacterial degradation of 2,6-dinitrotoluene. Appl. Environ. Microbiol. **66:**2139-2147. http://dx.doi.org/10.1128/aem.66.5.2139-2147.2000
2. **Sambrook J, Russell DW.** 2001. Molecular cloning: a laboratory manual, 3rd ed. Cold Spring Harbor Laboratory, Cold Spring Harbor.
3. **Herrero M, de Lorenzo V, Timmis KN.** 1990. Transposon vectors containing non-antibiotic resistance selection markers for cloning and stable chromosomal insertion of foreign genes in Gram-negative bacteria. J. Bacteriol. **172:**6557-6567.
4. **de Lorenzo V, Timmis KN.** 1994. Analysis and construction of stable phenotypes in gram-negative bacteria with Tn*5*- and Tn*10*-derived minitransposons. Methods Enzymol. **235:**386-405. http://dx.doi.org/DOI:10.1016/0076-6879(94)35157-0
5. **Bagdasarian M, Lurz R, Rückert B, Franklin FCH, Bagdasarian MM, Frey J, Timmis KN.** 1981. Specific purpose plasmid cloning vectors. II. Broad host range, high copy number, RSF1010-derived vectors, and a host-vector system for gene cloning in *Pseudomonas*. Gene **16:**237-247. http://dx.doi.org/10.1016/0378-1119(81)90080-9
6. **Martínez-García E, Jatsenko T, Kivisaar M, de Lorenzo V.** 2014. Freeing *Pseudomonas putida* KT2440 of its proviral load strengthens endurance to environmental stresses. Environ. Microbiol. **17:**76-90. http://dx.doi.org/10.1111/1462-2920.12492
7. **Choi KH, Gaynor JB, White KG, López C, Bosio CM, Karkhoff-Schweizer RR, Schweizer HP.** 2005. A Tn*7*-based broad-range bacterial cloning and expression system. Nat. Methods **2:**443-448. http://dx.doi.org/10.1038/nmeth765
8. **Zobel S, Benedetti I, Eisenbach L, de Lorenzo V, Wierckx N, Blank LM.** 2015. Tn*7*-based device for calibrated heterologous gene expression in *Pseudomonas putida*. ACS Synth. Biol. **4:**1341-1351. http://dx.doi.org/10.1021/acssynbio.5b00058
9. **Amann E, Ochs B, Abel KJ.** 1988. Tightly regulated *tac* promoter vectors useful for the expression of unfused and fused proteins in *Escherichia coli*. Gene **69:**301-315. http://dx.doi.org/10.1016/0378-1119(88)90440-4
10. **Vemuri GN, Altman E, Sangurdekar DP, Khodursky AB, Eiteman MA.** 2006. Overflow metabolism in *Escherichia coli* during steady-state growth: transcriptional regulation and effect of the redox ratio. Appl. Environ. Microbiol. **72:**3653-3661. http://dx.doi.org/10.1128/AEM.72.5.3653-3661.2006
11. **Benedetti I, Nikel PI, de Lorenzo V.** 2016. Data on the standardization of a cyclohexanone-responsive expression system for Gram-negative bacteria. Data in Brief **6:**738-744. http://dx.doi.org/10.1016/j.dib.2016.01.022
12. **Benedetti I, de Lorenzo V, Silva-Rocha R.** 2012. Quantitative, non-disruptive monitoring of transcription in single cells with a broad-host range *GFP-luxCDABE* dual reporter system. PLoS One **7:**e52000. http://dx.doi.org/10.1371/journal.pone.0052000
13. **Silva-Rocha R, Martínez-García E, Calles B, Chavarría M, Arce-Rodríguez A, de las Heras A, Páez-Espino AD, Durante-Rodríguez G, Kim J, Nikel PI, Platero R, de Lorenzo V.** 2013. The Standard European Vector Architecture (SEVA): a coherent platform for the analysis and deployment of complex prokaryotic phenotypes. Nucleic Acids Res. **41:**D666-D675. http://dx.doi.org/10.1093/nar/gks1119
14. **Nikel PI, Pérez-Pantoja D, de Lorenzo V.** 2016. Pyridine nucleotide transhydrogenases enable redox balance of *Pseudomonas putida* during biodegradation of aromatic compounds. Environ. Microbiol. **18:**3565-3582. http://dx.doi.org/10.1111/1462-2920.13434
15. **Svenningsen NB, Pérez-Pantoja D, Nikel PI, Nicolaisen MH, de Lorenzo V, Nybroe O.** 2015. *Pseudomonas putida* mt-2 tolerates reactive oxygen species generated during matric

stress by inducing a major oxidative defense response. BMC Microbiol. **15:**202. http://dx.doi.org/10.1186/s12866-015-0542-1
