# Supplementary material for: The Metabolic Redox Regime of Pseudomonas putida Tunes Its Evolvability toward Novel Xenobiotic Substrates
Source: mBio. 2018 Aug 28;9(4):e01512-18. doi: 10.1128/mBio.01512-18 (PMC6113623; doi:10.1128/mBio.01512-18)
Supplement: TEXT S1 [file mbo004184023s1.pdf]

Supplemental Materials and Methods

Strain and pathway engineering. A derivative of *Pseudomonas putida* strain EM173 (1) carrying different parts of the 2,4-dinitrotoluene (2,4-DNT) degradation pathway of *Burkholderia* sp. R34 was constructed as follows. In the first step, the 2,4-DNT pathway genes were amplified from strain R34 by PCR: *dntR* and $dntA_{abcd}$ genes (6.2 kb) were amplified with primers *RA*–F (5'-AGC TGA ATT CTT GCT GGA CGC TTA TGC T-3') and *RA*–R (5'-TTA ATC TAG ACC AGG GCT CAC AGG AAG AT-3'), *dntDEG* genes (~4.5 kb) were amplified with primers *DEG*–F (5'-TTA ATC TAG ACT GAT TCG TTC TCG CTC CAT-3') and *DEG*–R (5'-AGC TAC TAG TGC CTC AAG TGA CCT CTT TGG-3'), and the *dntB* gene (~1.8 kb) was amplified with primers *B*–F (5'-GAA CTC TAG AAA CGA GAC GGC ACG ATT TT-3') and *B*–R (5'-TTA ATC TAG ACT GGG AAG AAC CAT TTA GGC-3'). These amplicons were obtained using high-fidelity proof-reading DNA polymerase (Phusion™, Thermo Fisher Scientific). PCRs were performed as per the following conditions: pre-denaturation at 98ºC for 2 min, and 30 cycles with denaturation at 98 ºC for 15 sec, annealing at 60ºC for 30 s, extension at 72ºC (for 3 min for 6.2 kb fragment, 2 min for 4.5 kb and 1 min for 1.8 kb) with a final extension at 72ºC for 10 min. Vector pTn7_MCS (Table S1) was digested with EcoRI and XbaI and then ligated with an amplicon that contained the *dntR* and *dntAaAbAcAd* genes, restricted with the same enzymes. After confirmation by sequencing, the resulting plasmid was cut with XbaI and SpeI and then was ligated with a DNA fragment that contained the *dntDEG* genes, previously restricted with XbaI and SpeI. Finally, the PCR product containing the *dntB* gene was digested with XbaI and cloned into the XbaI site of the vector already containing the *dntR*, *dntAaAbAcAd*, and *dntDEG* genes. The final plasmid obtained by means of this procedure was termed pTn7·DNT (Table S1). After transformation of the ligation mixture in chemically-competent *Escherichia coli* CC118λ*pir*, clones were checked by colony PCR using *GoTaq* DNA polymerase and combinations of the primers described above, and later confırmed by DNA sequencing.

Conjugative matings were set using *E. coli* CC118λ*pir* (carrying plasmid pTn7·DNT) as the donor strain, the helper strain *E. coli* HB101 (carrying plasmid pRK600), and *P. putida* EM173 as the recipient strain (see Table S1 for details on strains and plasmids). Plasmid pTn7·DNT was co-mobilized along with the transposase-encoding genes *tnsABCD* into the recipient strains by including *E. coli* CC118λ*pir* carrying plasmid pTNS1 in the mating mixture. Conjugative matings were performed as described elsewhere (2, 3). Transconjugants were tested for their resistance to kanamycin. Only clones that were sensitive to kanamycin (and resistant to gentamicin) represent authentic transposition events and not integration of the whole plasmid into the chromosome. Insertion of the *dnt* genes in the resulting strain (*P. putida* EM·DNT) was further confirmed by colony PCR as indicated above. Furthermore, to verify the correct insertion of the transposon into the *att*·Tn*7* site in the bacterial chromosome, some clones resistant to gentamycin were selected and checked *via* Tn*7*-specific colony PCR. The correct targeting of the mini-Tn*7*[$dntA_{abcd}$*BDEG*] element to the *att*·Tn*7* site in *P. putida* EM·DNT was diagnosed with primers Tn7-2–F (5'-CCA CGC CCC TCT TTA ATA C-3') and PpuglmS-2–R (5'-GTG CGT GCC CGT GGT GG-3') or PpuglmS-5–R (5'-CGC AAG CTG CCG ACC TGT-3'), which generate amplification products of approximately 500 bp and 1 kb, respectively (4).

Construction of transcriptional fusions. Transcriptional fusions for *in vivo* assessment of the SOS response were constructed by directional cloning of PCR-amplified promoter regions from *P. putida* KT2440 into the multiple cloning site of the promoter-less *GFP-luxCDABE* vector pGLR2 (5). PCR primer pairs for the amplification of the *recA* promoter region were designed by including either BamHI or EcoRI recognition sequence extensions. The primers used for this

purpose were P_{recA}–F (5'-ATC GGA ATT CGT GGC GGT GAG TGG TGT G-3') and P_{recA}–R (5'-AGT CGG ATC CAA TCC TCA CGT GTT CGA CTT G-3'). Sequences were amplified from genomic DNA of *P. putida* KT2440 using high-fidelity proof-reading DNA polymerase (Phusion™). PCR was performed with the following conditions: pre-denaturation at 98ºC for 2 min, and 30 cycles with denaturation at 98ºC for 10 sec, annealing at 60 ºC for 30 s, extension at 72 ºC for 30 s with a final extension at 72 ºC for 10 min. Purified PCR products and vector were digested with BamHI and EcoRI; aliquots of these restrictions were ligated before transformation into *E. coli* DH5α competent cells. Selection of transformants carrying the appropriate plasmid was initially made on the basis of kanamycin resistance depending on the vector. The positive transformants were confirmed by colony PCR. Colony PCR amplification was performed by using the same forward and reverse primers used to amplify the *recA* promoter. The PCR was performed as the following conditions: pre-denaturation at 95 ºC for 2 min, and 30 cycles with denaturation at 95 ºC for 30 s, annealing at 60 ºC for 30 s, extension at 72 ºC for 1 min with a final extension at 72 ºC for 10 min. Plasmid preparations of colonies that resulted from ligation were prepared for sequencing and the final construct was verified by DNA sequencing. Plasmids pSR·*ahpC* and pSR·*katA*, containing the promoter region of *ahpC* and *katA*, respectively, cloned before the *msf·GFP* gene, were described before (6, 7), and were used to analyze the stress response of *P. putida* strains. All plasmids containing these transcriptional fusions were transferred into *P. putida* EM·DNT strain by electrotransformation (8).

Construction of a synthetic *NADH burning* device based on NADH oxidase. The gene encoding NADH oxidase from *Streptococcus pneumoniae* strain Cp1015 was excised from plasmid pTrc99A-nox (9) by double digestion with BamHI and PstI. The resulting 1.4-kb DNA fragment, encompassing *nox* along with an artificial ribosome binding site, was sub-cloned into the expression vector pSEVA2311 (10, 11) restricted with the same endonucleases. This operation gave rise to plasmid pS2311·Nox, in which the expression of *nox* is driven by a ChnR/P_{chnB} element. This plasmid was transferred to the *P. putida* strains indicated in the main text, and, whenever needed, the expression of *nox* was induced by addition of cyclohexanone at 0.1 mM at the onset of the cultivation.

Intracellular measurement of reactive oxygen species (ROS). The ROS sensitive green fluorescent dye 2',7'-dichlorodihydrofluorescein diacetate (H_2DCF-DA [Sigma-Aldrich Co.]) was used to quantitate ROS formation in *P. putida*. Cells incubated with H_2O_2 at 1 mM were included as a positive control in all the experiments (data not shown). After being incubated overnight in the corresponding culture medium, cells from 1-ml culture aliquots were pelleted by centrifugation at 8,000×*g* for 5 min, washed once with phosphate-buffered saline (PBS, pH= 7.5), and resuspended in PBS to adjust the optical density at 600 nm (OD_{600}) to ca. 0.4. This cell suspension was added with H_2DCF-DA to a final concentration of 20 μM, and then incubated in the dark for 30 min at room temperature. After the incubation period, cells were washed twice with PBS to remove any unbound label, resuspended in PBS, and immediately analyzed by flow cytometry. The H_2DCF-DA fluorescence emission at 525 nm was detected using a 530/30-nm band pass filter array.

Measurement of DNA damage elicited by 2,4-DNT *via* assessment of induction of the SOS response. For plate assays using strain EM·DNT carrying plasmid pGR·*recA*, a 100-μl aliquot of an overnight culture was inoculated into soft agar [0.7% (wt/vol) agar] and spread onto an M9 minimal medium plate containing 0.4% (wt/vol) succinate as the carbon source. A 2,4-DNT (0.5 M)–containing filter disc was placed onto the agar and the plate was visualized through a blue-light lamp after 24 h of incubation at 30°C. A disc with norfloxacin, a known inducer of the SOS system, was used as a positive control. To measure the impact of 2,4-DNT exposure on SOS pathway expression, we measured the GFP output in the EM·DNT carrying plasmid pGR·*recA* by using flow

cytometry. Overnight cultures were grown in M9 medium with 0.4% (wt/vol) succinate to exponential phase as indicated before and exposed to 2,4-DNT, H_2O_2, or norfloxacin. Cells were grown and treated with 2,4-DNT and/or other additives as explained in the text. Cells from 1-ml culture aliquots were pelleted by centrifugation at 8,000×*g* for 5 min, washed once with PBS, pH = 7.5, and resuspended in PBS to adjust the OD_{600} to ca. 0.4. This cell suspension was added with H_2DCF-DA at 20 μM from a freshly-prepared stock solution in DMSO, and then incubated in the dark for 30 min at room temperature. After the incubation period, cells were washed once with PBS to remove unbound label, resuspended in PBS, and immediately analyzed by flow cytometry as indicated above.

Flow cytometry. Single-cell fluorescence was analyzed with a MACSQuant VYB flow cytometer (Miltenyi). GFP was excited at 488 nm, and the fluorescence signal was recovered with a 525/40 nm band pass filter. Cells were harvested when cultures reached mid-exponential phase (OD_{600} = 0.5) and at least 15000 events were analyzed for every aliquot. The induction of bioreporters under the conditions tested was quantified by firstly gating the cells in a side scatter against forward scatter plot, and then the GFP-associated fluorescence was recorded in the FL1 channel (515-545 nm). Data processing was performed using the FlowJo™ software (12).

Sequencing of the *rpoB* locus in *P. putida*. An internal fragment of the *P. putida rpoB* sequence was amplified with primers *Pp·rpoB*–F (5'-GGC GGA AAG CGA AGG CCT GAT G-3'), complementary to the nucleotide sequence at positions 1,458-1,479, and *Pp·rpoB*–F (5'-CAA CGC CTT CCT TCA CCA CGC GG-3'), complementary at positions 1,788-1,810 relative to the ATG initiation codon of the *rpoB* gene of *P. putida*, essentially as indicated elsewhere (13). The resulting 354-bp long amplicon was sequenced, and the BioEdit™ and MultAlign™ software were used for sequence comparison. In some cases, in which the sequencing results were not completely clear, primers *Pp·rpoB2*–F (5'-GAC CCT GGT CGA TAT CCG TA-3') and *Pp·rpoB2*–R (5'-GCT CCA TGC CGG TAC CTA-3') were used to amplify a 827-bp long region between coordinates 1,305 and 2,131 nt of the *rpoB* gene, and primers *Pp·rpoB*–F and *Pp·rpoB*–F were then used for sequencing of the resulting amplicon.

Other analytical procedures. For identification of intermediate metabolites from the 2,4-DNT degradation pathway, overnight-grown *P. putida* EM·DNT cells were used as the inoculum by diluting them to a starting OD_{600} of ca. 0.05. Cells were grown in the same culture medium until they reached an OD_{600} of ca. 0.5, at which point the cell suspension was split into two 125-ml Erlenmeyer flasks, one culture served as a control experiment and was added with DMSO as a control and the other one was added with DNT to a final concentration of 0.5 mM. Cells were aerobically grown overnight at 30°C and then removed by centrifugation at 3,000×*g*, 15 min at 4°C and the supernatant kept for analysis of DNT biodegradation intermediates. Metabolites were extracted from the biomass and the culture supernatants by using either $CHCl_3$ or ethyl acetate. Accumulation of the yellow intermediate 4-methyl-5-nitrocatechol was spectrophotometrically quantified by absorbance readings at 420 nm and by gas chromatography-mass spectrometry (14). Total NADH oxidase activity was measured spectrophotometrically by monitoring the initial rate of decrease in NADH absorbance at 340 nm and at 30°C in the soluble fraction obtained from a total cell-free extract as reported elsewhere (6). One unit of NADH oxidase activity corresponds to the oxidation of 1 μmol of NADH per min, using a molar extinction coefficient for NADH (ε_{NADH}) of 6.22 mM^{-1} cm^{-1} at 340 nm. The intracellular NADH and NAD^+ concentrations were obtained using *in vitro* cyclic assays (15).

REFERENCES

1. **Martínez-García E, Jatsenko T, Kivisaar M, de Lorenzo V.** 2014. Freeing *Pseudomonas putida* KT2440 of its proviral load strengthens endurance to environmental stresses. Environ. Microbiol. **17:**76-90. http://dx.doi.org/10.1111/1462-2920.12492
2. **Martínez-García E, Aparicio T, de Lorenzo V, Nikel PI.** 2014. New transposon tools tailored for metabolic engineering of Gram-negative microbial cell factories. Front. Bioeng. Biotechnol. **2:**46. http://dx.doi.org/10.3389/fbioe.2014.00046
3. **Martínez-García E, de Lorenzo V.** 2012. Transposon-based and plasmid-based genetic tools for editing genomes of Gram-negative bacteria. Methods Mol. Biol. **813:**267-283. http://dx.doi.org/10.1007/978-1-61779-412-4_16
4. **Choi KH, Schweizer HP.** 2006. Mini-Tn*7* insertion in bacteria with single *att*Tn*7* sites: example *Pseudomonas aeruginosa*. Nat. Protoc. **1:**153-161.
5. **Benedetti I, de Lorenzo V, Silva-Rocha R.** 2012. Quantitative, non-disruptive monitoring of transcription in single cells with a broad-host range *GFP-luxCDABE* dual reporter system. PLoS One **7:**e52000. http://dx.doi.org/10.1371/journal.pone.0052000
6. **Nikel PI, Pérez-Pantoja D, de Lorenzo V.** 2016. Pyridine nucleotide transhydrogenases enable redox balance of *Pseudomonas putida* during biodegradation of aromatic compounds. Environ. Microbiol. **18:**3565-3582. http://dx.doi.org/10.1111/1462-2920.13434
7. **Svenningsen NB, Pérez-Pantoja D, Nikel PI, Nicolaisen MH, de Lorenzo V, Nybroe O.** 2015. *Pseudomonas putida* mt-2 tolerates reactive oxygen species generated during matric stress by inducing a major oxidative defense response. BMC Microbiol. **15:**202. http://dx.doi.org/10.1186/s12866-015-0542-1
8. **Choi KH, Kumar A, Schweizer HP.** 2006. A 10-min method for preparation of highly electrocompetent *Pseudomonas aeruginosa* cells: application for DNA fragment transfer between chromosomes and plasmid transformation. J. Microbiol. Methods **64:**391-397. http://dx.doi.org/10.1016/j.mimet.2005.06.001
9. **Vemuri GN, Altman E, Sangurdekar DP, Khodursky AB, Eiteman MA.** 2006. Overflow metabolism in *Escherichia coli* during steady-state growth: transcriptional regulation and effect of the redox ratio. Appl. Environ. Microbiol. **72:**3653-3661. http://dx.doi.org/10.1128/AEM.72.5.3653-3661.2006
10. **Benedetti I, de Lorenzo V, Nikel PI.** 2016. Genetic programming of catalytic *Pseudomonas putida* biofilms for boosting biodegradation of haloalkanes. Metab. Eng. **33:**109-118. http://dx.doi.org/10.1016/j.ymben.2015.11.004
11. **Benedetti I, Nikel PI, de Lorenzo V.** 2016. Data on the standardization of a cyclohexanone-responsive expression system for Gram-negative bacteria. Data in Brief **6:**738-744. http://dx.doi.org/10.1016/j.dib.2016.01.022
12. **Nikel PI, Romero-Campero FJ, Zeidman JA, Goñi-Moreno A, de Lorenzo V.** 2015. The glycerol-dependent metabolic persistence of *Pseudomonas putida* KT2440 reflects the regulatory logic of the GlpR repressor. mBio **6:**e00340-00315. http://dx.doi.org/10.1128/mBio.00340-15
13. **Jatsenko T, Tover A, Tegova R, Kivisaar M.** 2010. Molecular characterization of Rif^R mutations in *Pseudomonas aeruginosa* and *Pseudomonas putida*. Mutat. Res. **683:**106-114. http://dx.doi.org/10.1016/j.mrfmmm.2009.10.015
14. **de las Heras A, Chavarría M, de Lorenzo V.** 2011. Association of *dnt* genes of *Burkholderia* sp. DNT with the substrate-blind regulator DntR draws the evolutionary itinerary of 2,4-dinitrotoluene biodegradation. Mol. Microbiol. **82:**287-299. http://dx.doi.org/10.1111/j.1365-2958.2011.07825.x
15. **Nikel PI, Chavarría M.** 2016. Quantitative physiology approaches to understand and optimize reducing power availability in environmental bacteria, p. 39-70. *In* McGenity TJ, Timmis KN,

and Nogales-Fernández B (ed.), Hydrocarbon and Lipid Microbiology Protocols–Synthetic and Systems Biology - Tools. Humana Press, Heidelberg, Germany. http://dx.doi.org/10.1007/8623_2015_84
